# Dr. Beddoes's Popular Essays

**Published:** 1801-10

**Authors:** T. Beddoes

**Affiliations:** Clifton


### *To the Editors of the Medical and Phyſical Journal.*

Gentlemen,

A Temporary inability to uſe my eyes freely, and ſome other circumſtances, oblige me to poſtpone my intended popular Eſſays to the beginning of December; after which, as far as depends on me, they will proceed regularly. I do not regret this delay, as it gives me the opportunity of addreſſing to practitioners of medicine, and to other obſervers of human life, the following requeſt: If they have any facts likely to promote the public health, whether reſpecting quack medicines, or of any other deſcription which they think ſuitable to my plan, I ſhould be much obliged by the communication of them. I could wiſh to be authorized to uſe the name of my correſpondent, as a voucher for the facts; but, at all events, I muſt be ſatisfied myſelf of their reality. I am, &c.

